# Minutes of the Medical Association of Georgia

**Published:** 1901-05

**Authors:** 


### SOCIETY REPORTS.

---

### MINUTES OF THE MEDICAL ASSOCIATION OF GEORGIA.

*Fifty-second Annual Session.*

FIRST DAY—MORNING SESSION.

AUGUSTA, GA., April 17, 1901.

The Association met in the City Court room of the Richmond county court-house, and was called to order by the president, Dr. S. C. Benedict of Athens, at 10 A.M.

Prayer was offered by the Rev. C. C. Williams, D.D., of Augusta; after which the Hon. Jacob Phinizy, mayor of Augusta, delivered an address of welcome on behalf of the city of Augusta. He was followed by Dr. J. B. Morgan of Augusta, who welcomed the Association on behalf of the medical profession of Augusta.

The president announced that the response to the addresses of welcome on behalf of the Medical Association was to have been delivered by Dr. Frank Ridley of LaGrange, but that word had been received from him that he would be unable to be present, and therefore the president called upon Dr. Baird, who responded in an eloquent and pleasing manner.

After this response the president stated that the "President's Annual Address" should have been omitted from the program, as it had been dispensed with some time since.

The president invited all ex-presidents to take seats upon the platform.

Dr. Eugene Foster, as chairman, presented the report of the Committee of Arrangements, which was adopted.

Dr. V. D. Lockhart, vice-president of the Association, was invited to occupy a seat upon the platform.

In the absence of Dr. St. J. B. Graham, chairman of the Program Committee, the report of this committee was made by Dr. Howard J. Williams. The report was adopted.

The president called for the report of the Committee on Publication, and the secretary stated that as an ex-officio member of the committee he would include the report of this committee in the secretary's report.

On account of the absence of the chairman of the Committee on Necrology, Dr. Williams moved that the reading of this report be postponed until the afternoon session. Motion carried.

Report of Committee on New Members was postponed.

Dr. Jas. B. Baird, as chairman, read the report of the Committee on Medical Legislation.

Dr. Hunter P. Cooper called attention to the valuable work which had been done by this committee in their untiring efforts in behalf of the Association and the people of Georgia, and stated that some permanent organization should be established for the opposition of osteopathists and others of this class.

The president called attention to the recommendation in the report for a charter for the Association.

Dr. Foster moved that the report of the committee be adopted and that Dr. Biard be requested to present the matter of a charter at the next day's session. Motion carried.

Dr. J. C. King introduced a resolution extending a vote of thanks to Senator Ellis and others for their work in opposing the osteopathy bill.

Attention was called to the fact that this was mentioned in the report of the Committee on Medical Legislation, and on motion this resolution was referred to this committee to report back to the Association.

On motion of Dr. Foster, a rising vote of thanks was unanimously extended to Dr. Baird and the other members of his committee for their thorough work.

The report of the Committee on Constitution and By-laws was postponed until later.

Dr. W. F. Westmoreland read the report of the Committee on New Members, which was adopted.

The president called attention to the fact that there were not sufficient members of the Board of Censors present to make a quorum, and therefore to facilitate the business of the Association would call for a vote upon the amendment to the Constitution, which was introduced at the meeting of the Association in 1900 and which had to lie upon the table for one year, and which resolution conferred upon the president the power to appoint temporary members on the Board of Censors sufficient to make a quorum.

Discussion upon this amendment developed considerable opposition to confining the power of the president to appointing simply a quorum instead of a full membership of the Board of Censors, and upon being put to a vote the amendment was lost.

Dr. Foster called attention to the fact that the president had the power to complete the Board of Censors by the precedent which had been established in the past, of appointing temporary members to serve until the regular members should appear.

Acting under the authority conferred by this precedent, the president appointed the following members to complete the Board of Censors: Dr. V. O. Hardon, to act as chairman in place of Dr. W. S. Elkin, and Dr. B. R. Doster in place of Dr. Chas. Hicks.

The President introduced Dr. Geo. R. Fowler of Brooklyn to the Association and he was invited to occupy a seat on the platform.

The reading of papers was proceeded with.

Dr. Howard J. Williams, of Macon, read a paper on "Excision in Tuberculosis of Joints, Hip and Wrist," and this was discussed by Drs. W. F. Westmoreland and Hunter P. Cooper.

Dr. W. F. Westmoreland, of Atlanta, read a paper on "Report of Surgical Cases," which was discussed by Drs. McRae, Earnest, Fowler and Westmoreland (in closing).

On motion the Association adjourned to meet at 3:30 P.M.

### FIRST DAY—AFTERNOON SESSION.

Association assembled at 3:30, and was called to order by the president.

The president read a telegram from Dr. A. W. Calhoun stating that at the last moment he was prevented from attending the meeting and sent his regrets.

Dr. Thomas R. Wright, of Augusta, read a paper entitled "Report of Surgical Cases." This was discussed by Dr. Earnest.

Dr. Harbin, of Rome, introduced a resolution on vaccination which was referred to the Committee on Medical Legislation.

Dr. Geo. R. Fowler, of Brooklyn, read a paper entitled "Internal Derangement of the Knee-joint, with report of three cases of Removal of the Internal Meniscus, or Semi-Lunar Cartilage."

Dr. Jno. G. Earnest, of Atlanta, read a paper on "Hysterectomy, with Interesting Complications."

Dr. P. L. Cortelyou, of Marietta, Ga., read a paper entitled "Report of Compound Fracture of the Lower Jaw."

Dr. E. C. Davis, of Atlanta, read a paper on "Cæsarean Section, with Report of Successful Operation on a Patient with a Rachitic Pelvis." This was discussed by Drs. Earnest and Ross.

Dr. D. A. N. Thomas, of Jersey, Ga., read a paper on "Lung Injuries."

Dr. W. H. Doughty, of Augusta, exhibited a specimen of a pregnant ovary, which was removed twelve months after gestation.

Dr. V. O. Hardon, of Atlanta, read a paper entitled "The Alum Enema in the After Treatment of Abdominal Operations," and this was discussed by Drs. Morgan, McRae and Earnest.

The President then called for report of special committees, as this closed the afternoon program of papers.

Dr. Baird, as chairman of the Committee on Medical Legislation, presented a resolution of thanks to the members of the General Assembly who had rendered such valuable aid in opposing the osteopathy bill. This was adopted.

Dr. Elliott, of Savannah, stated that he had to leave the city that night, and as he came to Augusta especially to invite the Association to meet next year in Savannah, that he would like to do so. He then extended a hearty invitation for the Association to come to Savannah, and urged that it be accepted.

The president stated that during the past year he had appointed Dr. J. H. Shorter of Macon a delegate to the Pan-American Congress which met in Havana.

On motion the Association adjourned to meet at 9:30, Thursday morning.

SECOND DAY—MORNING SESSION.

The Association met at 9:30 A.M., and was called to order by the president.

The reading of the minutes of the previous sessions were dispensed with.

The report of the secretary, Dr. Louis H. Jones, was read and adopted.

In this report the secretary stated that he had on hand a large number of back numbers of the Transactions of the Association, and requested that he be instructed to save only a certain number of each year, and to destroy the balance.

After a discussion as to the disposition to make of these back numbers, it was finally decided that the Transactions were too valuable to be destroyed, and that the secretary notify the members of the Association that all who did not have complete files of the Transactions could complete their files by at once notifying the secretary, and that all copies which were left over at the end of ninety days, after completing the files of the secretary, could be destroyed.

The president then called for reports from special committees, and Dr. Baird presented the following resolution in regard to securing a charter for the Association:

"*Resolved*, That the Committee on Medical Legislation be instructed to procure for this Association a suitable charter, and that the sum of fifty dollars, or so much thereof as necessary, be appropriated to cover the expense incident thereto."

The resolution was adopted.

Dr. Henry R. Slack, as chairman, read the report of the Committee on Pasteur Institute, as follows:

REPORT OF COMMITTEE ON PASTEUR INSTITUTE.

*Mr. President and Members of the Medical Association of Georgia:*

Your committee met in Atlanta, May 1, 1900, in Dr. Hurt's office and organized, electing Dr. Claude A. Smith secretary.

We issued the following letter to twenty-five physicians in different parts of the State, inviting them to become charter members.

LAGRANGE, GA., May 10th, 1900.

DEAR DOCTOR :—At the meeting of the Medical Association of Georgia in Atlanta last month a paper was read showing the necessity for a Pasteur Institute in Georgia. After the discussion a committee was appointed to investigate the matter, "with power to act," and the following resolutions passed:

*Resolved*, That the Medical Association of Georgia indorse the establishment of a Pasteur Institute and Laboratory in Georgia.

*Resolved 2d*, That we will endeavor to secure, through legislative enactment, 50 per cent. of the tax on dogs for the support of said intitution.

The committee met in Atlanta, May 1st, and decided it would be better to establish the Pasteur Institute and Laboratory in a home of its own.

We are going to apply for a charter June 1st, and would like to have you as a CHARTER MEMBER. The membership fee will be ten ($10.00) dollars and no dues. The fee will include a handsome lithograph certificate of membership. If the institute pays you will receive dividends on your stock. You will also receive a discount of 50 per cent. from regular prices for all pathological and bacteriological work done in the laboratory.

Enclosed you will find a list of those you will be associated with as charter members.

Please let me hear from you at once, or before the 28th, as our committee meets to make application for charter on 31st.

Yours fraternally,

HENRY R. SLACK, Chairman.

Seventeen replied favorably, but five failed to respond with the $10.00 fee. The deficit was made up by the remaining twelve, and on May 31st, we had an enthusiastic meeting in Atlanta, and employed Messrs. Kontz & Austin to obtain a charter for the Georgia Pasteur Institute and Laboratory. They promptly drew us a very liberal and satisfactory charter, but owing to Judge Lumpkin's absence in Europe, the same was not signed until September 8th, 1900. We then issued the following letter:

LAGRANGE, GA., Sept. 5th, 1900.

DEAR DOCTOR :—I am delighted to inform you that our attorneys, Messrs. Kontz & Austin, have advised me that the "charter for the Pasteur Institute and Laboratory will be signed, sealed and delivered on the 8th inst."

I will therefore call a meeting of the committee, charter members and original subscribers for September 11th, 1 P.M., in Dr. Hurt's office, Equitable Building, Atlanta. At this meeting we wish to organize, elect board of directors, formulate plans for management, select site, place stock and make necessary arrangements to start to work, for there is plenty of it to do.

Please try to be on hand, but if it is impossible, send your proxy to some member of the committee, for we want to be operating before the legislature meets.

Hoping to see you next Tuesday, I am,

Yours fraternally,

HENRY R. SLACK, Chairman.

In response to it a majority of the committee met September 11th, subscribed $1,000 stock and proceded to organize by adopting a constitution and by-laws and electing the following board of governors: Drs. C. D. Hurt, Jas. N. Brawner, Claude A. Smith, J. H. McDuffie, E. P. Ham, St. J. B. Graham, Henry R. Slack, and Messrs. B. M. Hunt and Geo. C. Walters.

The board of governors then elected as officers, president, Henry R. Slack, M.D.; vice-president, J. H. McDuffie, M.D.; treasurer, C. D. Hurt, M.D.; secretary and pathologist, Claude A. Smith, M.D.; physician-in-charge, Jas. N. Brawner, M.D.

We rented a house, No. 34 Auburn Ave., Atlanta, and on November 15th were ready for patients. The Pasteur Institute is now well equipped and has a pathological and bacteriological laboratory, modest in proportions, but thorough in its work.

We have treated eleven persons bitten by rabid animals (eight proved to be rabid by inoculations of rabbits), all of whom are now well and grateful to this association for encouraging the establishment of a Pasteur Institute in the South.

We have also turned away a number who applied, but there was no evidence to show that they were bitten by rabid animals.

At the last meeting of the board of governors every member of this Association in good standing was elected a fellow of the Pasteur Institute, and upon payment of $10 you will receive a certificate and one share of stock. Fellowship entitles you to a reduction of 50 per cent. on all pathological work you have done in the laboratory and will pay a handsome dividend on your stock if you only have one examination made a year. Your stock will also share in any profit made in the Pasteur department. Our financial report for five months is as follows:

| Georgia Pasteur Institute and Laboratory | Dr. | Cr. |
|---|---|---|
| Capital stock paid in | $1,000 00 | |
| Received for treatment and pathology work | 1,110 50 | |
| Cr. | | |
| Charter and apparatus | | 1,000 00 |
| Salary of physician and pathologist | | 350 00 |
| Running expenses | | 523 15 |
| Cash, notes and accounts | | 237 35 |
| | $2,110 50 | $2,110 50 |

This shows a profit of over 20 per cent., although we have treated nearly one-fifth of the cases free.

We would like to have you all take at least one share of stock for $10 and pay it now to our secretary, Dr. Claude A. Smith. We urge this for three reasons: (1) We want your financial backing so we can erect a suitable building—a home that each fellow can point to with pride. (2) The more members we have the greater our usefulness (we don't want a small close corporation). (3) It is a good investment for you, as it has now passed through the experimental stage, and we believe dividends are assured.

We hope the action of your committee will meet with your approval and will receive both your moral and financial support.

Respectfully submitted,

HENRY R. SLACK, Chairman,
C. D. HURT,
CLAUDE A. SMITH,
J. H. MCDUFFIE.

Dr. Foster moved that this report be referred to a special committee for consideration, and that they report back to the Association. Motion carried.

The president appointed on this committee Drs. Foster, Powell, Holt, Doster and Baird.

Dr. Louis H. Jones moved that section 5, article 7 of the By-Laws, which reads, "No member shall receive a copy of the Transactions until his fees are paid in full," be stricken out. Motion carried.

Dr. Jones read a letter from the Dooly County Society of Medicine appointing delegates to the State Association.

Dr. Dunbar Roy, of Atlanta, read a paper on "Use and Abuse of Nasal Sprays," and this was discussed by Drs. Hobbs, Cox, Peete, and Roy in closing.

Dr. J. Lawton Hiers, of Savannah, presented a paper on "Protargol, its Efficacy in Ophthalmia Neonatorum and Trachoma."

This paper was discussed by Drs. Roy, Cortelyou, Kime and Hiers.

Dr. Harbin introduced the following resolution:

*Resolved*, That the Medical Association of Georgia commend the recent action of the legislature in authorizing the appointment of an ear, eye, nose and throat specialist to the Georgia School for the Deaf at Cave Spring, as a wise measure, and which cannot fail to contribute benefit to the institution.

Adopted.

Dr. Ross P. Cox, of Rome, read a paper on "A Contribution to the Study of Deafness."

Dr. L. J. Sharp, of Harmony Grove, contributed a paper on "Epidemic Sore Throat."

Dr. M. L. Currie, of Savannah, presented a paper on "Ophthalmia Neonatorum from the Standpoint of the General Practitioner."

Dr. Arthur W. Hobbs, of Atlanta, read a paper on "The Baneful Effects and Curability of Catarrhal Inflammation of the Upper Air Passages."

The time for adjournment being near at hand the discussion of the last four papers was postponed until the evening session.

Dr. Holt, as chairman of the Committee on Necrology, read the report of that committee, showing that the Association had lost three honorary and eight active members during the past year.

On account of the barbecue to be given to the Association in the afternoon, the meeting adjourned until 8 P.M.

SECOND DAY—NIGHT SESSION.

The Association met at 8 P.M., and was called to order by the president.

The president announced that the Georgia Pharmaceutical Association had elected three delegates to the Medical Association of Georgia with a view to encouraging friendly relations between the two associations and also for business purposes, and that Dr. Geo. F. Payne, of Atlanta, as one of those delegates was present and would state the wishes of the Pharmaceutical Association.

The President then called upon Dr. Payne and he stated that the Pharmaceutical Association wished the Medical Association to appoint delegates to represent them at the meeting of the Pharmaceutical Association and that the latter would do the same for the Medical Association, and thus promote a better relationship between the two Associations. Also, that as there were a number of different formulæ for certain preparations, that the Medical Association select a committee to meet with a similar committee from the Pharmaceutical Association to adopt standard formulæ for these preparations.

It was moved that the Association appoint three delegates to attend the meeting of the Pharmaceutical Association and that they report back to the Georgia Medical Association in regard to adopting standard formulæ for certain preparations.

This motion was seconded and carried and the president appointed the following delegates to the Georgia Pharmaceutical Association: Dr. H. R. Slack, Dr. K. P. Moore and Dr. V. O. Hardon.

Dr. J. H. Bradley, of Dublin, read a paper on "The Importance of Proper Management of the Third Stage of Labor: The Cause and Treatment of Some Forms of Hemicrania," which was discussed by Dr. L. H. Jones.

Dr. S. A. Visanska, of Atlanta, contributed a paper on "Infant-Feeding," and exhibited apparatus for preparing the food.

Dr. Wm. Z. Holliday, of Augusta, presented a paper entitled "Bottle-fed Babies."

Dr. Adair, of Harmony Grove, presented a paper on "General Hygiene of the Teeth."

Dr. M. B. Hutchins, of Atlanta, read a paper on "The treatment of External Cancer with Caustic Potash."

On motion, the Association adjourned until 9:30 A.M. Friday.

### THIRD DAY—MORNING SESSION.

The Association reassembled at 9:30 and was called to order by the president.

On account of the previous night's session the reading of the minutes was dispensed with.

Dr. J. Lawton Hiers read the report of the committee which was continued from last meeting of the Association and which had been previously appointed to confer with the medical societies of other States, inviting their cooperation in a memorial to Congress, asking an increase in the rank, pay, and allowance of the surgeon-general of the United States army. The report stated that favorable action had been taken by the American Medical Association and also in many other States by the different medical societies and associations, that a bill had been introduced in Congress, and that with the united efforts on the part of the medical men, hoped to have definite action taken on same at the next session of Congress.

Dr. Kime moved that the report of the committee be adopted and that they be reimbursed for the expenses incurred. Motion carried.

Dr. T. O. Powell, of Milledgeville, read a paper entitled "Marriage and Heredity; Its Relation to Insanity."

As the next two papers were along the same line as this one, it was decided to postpone the discussion until all had been read and then discuss them together.

Dr. W. L. Champion, of Atlanta, read a paper on "Venereal Diseases; Their Prevention."

Dr. R. R. Kime, of Atlanta, presented a paper on "Some Social Problems."

These papers were discussed by Drs. Baird, Williams, Hardeman, Spratling, Chason, and Drs. Champion and Kime (in closing).

Dr. Michael Hoke, of Atlanta, read a paper entitled "Pronated Foot," and exhibited apparatus to demonstrate treatment.

The president then announced that the report of the treasurer would be read.

Dr. Goodrich, as treasurer, read his report, and this was followed by a lively discussion which lasted until one o'clock, the hour set aside by the Constitution for the election of officers, and as nothing definite had been decided upon, the further consideration of the treasurer's report was postponed.

Before proceeding with the election of officers, the president announced that it would be in order to vote upon the adoption of the amendment to section 1, article 3 of the Constitution, which was introduced at the last meeting of the Association, and which provided for the consolidation of the offices of secretary and treasurer.

This amendment was adopted, and the election of officers was then taken up and resulted as follows:

President, Dr. Jas. B. Baird, Atlanta.
First Vice-President, Dr. Thos. R. Wright, Augusta.
Second Vice-President, Dr. J. D. Chason, Bainbridge.
Secretary and Treasurer, Dr. Louis H. Jones, Atlanta.
Censor, Dr. W. H. Doughty, Jr., Augusta.
The Association adjourned until 3:30 P.M.

AFTERNOON SESSION—THIRD DAY.

Dr. J. C. King introduced the following resolution, which was unanimously adopted:

*Resolved*, That the Medical Association of Georgia extend to Dr. Benedict a vote of thanks for the able manner in which he has presided over the sessions of the Association.

The president called for the report of the Auditing Committee, and Dr. Westmoreland, as chairman of that committee, stated that they had not yet reached any conclusion with the treasurer, but that if the Association would give them more time and sufficient latitude to cover the case that they could reach a settlement.

Dr. Louis H. Jones introduced the following: That the hearty thanks of the Association be tendered the Auditing Committee for their very efficient and faithful services during the past year; that the committee be continued with full power to act, and that such settlement as they see fit to make between the Association and the treasurer, Dr. Goodrich, will be entirely satisfactory to the Association.

This was seconded and carried.

Dr. J. B. Morgan moved that the Auditing Committee be reimbursed for any expenses they may have incurred. Carried.

Dr. J. Lawton Hiers extended a hearty invitation in behalf of the Georgia Medical Society of Savannah for the Association to meet at that place next year.

The president announced that the medical profession of Rome, Ga., had also extended a cordial invitation for the Association to meet at that place.

On motion of Dr. Westmoreland, the Association selected Savannah as the next place of meeting.

Dr. Foster moved that the Auditing Committee be allowed to make their report after the sessions of the Association so as to have it in the Transactions. Motion was carried.

Dr. Hardon read the report of the Board of Censors, in which they recommended that the Association appropriate $50.00 for the use of the Committee on Medical Legislation. This was put in the form of a motion and carried.

Dr. Foster called attention to the fact that the president-elect, Dr. Baird, was chairman of the Committee on Medical Legislation, and in view of the fact that he did not know of any man who could supplant Dr. Baird, he moved that Dr. Baird be continued as ex-officio chairman of the Committee on Medical Legislation. Motion carried.

Dr. Westmoreland moved that the secretary and treasurer, Dr. Jones, be allowed to settle such vouchers as had been signed by the president, Dr. Benedict, but which had not been paid by the treasurer, Dr. Goodrich. Motion carried.

### INSTALLATION OF OFFICERS.

In retiring, Dr. Benedict thanked the chairmen of the various committees for the valuable assistance which they had rendered, calling special attention to the work of Dr. Baird as chairman of the Committee on Medical Legislation. He then introduced Dr. Baird, who was escorted to the chair amid applause.

The reading of papers was taken up.

The next paper on the program was by Dr. T. E. Oertel of Augusta, entitled "Yellow Atrophy of Liver, with Report of a Case."

Dr. Oertel stated that as the time was short, and there were several present who had come a long distance to read their papers, that he would simply read his paper by title.

Dr. Henry R. Slack, of LaGrange, read a paper on "Hydrophobia; Its Prevalence and Prevention."

This paper was discussed by Drs. Foster, Westmoreland, Graham, Spratling and Claude A. Smith.

Dr. James T. Ross, of Macon, read a paper entitled "Tetanus—Report of Case; Intubation." This was discussed by Drs. Graham, Holt, Doster and Jones.

Dr. T. Virgil Hubbard, of Atlanta, presented a paper on "The Antiseptic and Eliminative Treatment of Typhoid Fever."

Dr. Foster moved that, on account of the lateness of the hour, the following papers be read by title. Motion carried.

"Types of Smallpox," by Dr. Eugene Foster of Augusta.

"Some Observations in a Series of Fifty Cases of Malaria," by Dr. E. E. Murphey of Augusta.

"Malarial Neuritis," by Dr. St. J. B. Graham of Savannah.

"A New Discovery in the Treatment of Chronic Diseases Dependent upon Cell Changes," by Dr. J. Cheston King of Atlanta.

Dr. Westmoreland moved that the secretary be paid an honorarium of $150.00. Motion carried.

Dr. Foster moved that the treasurer be paid an honorarium of $150.00. After some discussion this was finally adopted.

Dr. Louis A. Jones moved that Dr. Claude A. Smith be selected as the official reporter for the Association. Carried.

Dr. Westmoreland moved that a vote of thanks be tendered the Medical Profession of Augusta for their generous entertainment of the Association, also the same be tendered the clubs and hotels.

The motion was unanimously carried.

Dr. Hiers introduced the following:

WHEREAS, The members of the Medical Association of Georgia are put to no little inconvenience in securing and having properly signed the certificates granting us one-third railroad fare; and

WHEREAS, Many of our members living in inaccessible parts of the State are not even able to secure the certificates from the station agent giving them the usual reduction, thereby forcing them to pay full fare, at least to some more central point; and

WHEREAS, These hardships operate in a manner to greatly reduce the attendance and materially lessen the scientific work which should be done at our meetings; be it

*Resolved first*, by the Medical Association of Georgia, That the Southeastern States Passenger Association be requested to grant to our members one fare for the round trip when attending the annual meetings of the Association.

*Resolved second*, That a special committee of three be appointed to carry out the purposes of the foregoing resolution.

Resolutions were adopted.

On motion the Association adjourned to meet the third Wednesday in April, 1902, at Savannah, Ga.

